# A Comprehensive Approach to Posttraumatic Lymphedema Surgical Treatment

**DOI:** 10.1055/s-0043-1768645

**Published:** 2023-08-02

**Authors:** Nicolás Pereira, Vanessa Oñate, Ricardo Roa

**Affiliations:** 1 Department of Plastic Surgery and Burns, Hospital del Trabajador, Santiago, Chile; 2 Specialized Center for Lymphedema and Lipedema, Clínica Nea, Santiago, Chile

**Keywords:** lymphedema, posttraumatic lymphedema, SCIP flap, lymphovenous anastomosis, supermicrosurgery

## Abstract

**Background** Posttraumatic lymphedema (PTL) is sparsely described in the literature. The aim of this study is to propose a comprehensive approach for prevention and treatment of PTL using lymphovenous anastomosis (LVA) and lymphatic vessels free flap, reporting our experience in the management of early-stage lymphedema.

**Methods** A retrospective observational study was performed between October 2017 and July 2022. Functional assessment with magnetic resonance lymphangiography and indocyanine green lymphography was performed. Patients with lymphedema and functional lymphatic channels were included. Cases with limited soft tissue damage were proposed for LVA, and those with acute or prior soft tissue damage needing skin reconstruction were proposed for superficial circumflex iliac artery perforator lymphatic vessels free flap (SCIP-LV) to treat or prevent lymphedema. Primary and secondary outcomes were limb volume reduction and quality of life (QoL) improvement, respectively. Follow-up was at least 1 year.

**Results** Twenty-eight patients were operated using this approach during the study period. LVA were performed in 12 patients; mean reduction of excess volume (REV) was 58.82% and the improvement in QoL was 49.25%. SCIP-LV was performed in seven patients with no flap failure; mean REV was 58.77% and the improvement QoL was 50.9%. Nine patients with acute injury in lymphatic critical areas were reconstructed with SCIP-LV as a preventive approach and no lymphedema was detected.

**Conclusion** Our comprehensive approach provides an organized way to treat patients with PTL, or at risk of developing it, to have satisfactory results and improve their QoL.

## Introduction

In developed countries, the most common cause of secondary lymphedema is lymphadenectomy and radiation for cancer treatment. Trauma is an important cause of extremity lymphedema; nevertheless, posttraumatic lymphedema is poorly understood and its incidence is not clear. Persistent edema of the limbs after trauma is estimated up to 20 to 25%; however, available evidence is focused on posttraumatic edema of the limbs and does not necessarily involve an injury of the lymphatic system. 1

The lymphatic system has a delicate balance, and under normal conditions, a functional system can match transport capacity and lymphatic load. Over time, if the load presented is higher than the transport capacity, either because of additional lymphatic load or a damaged, attenuated lymphatic system, lymphedema will occur. In lower and upper extremities, the deep and superficial lymphatic system should not be considered as independent systems, since pathology in one can affect the other. However, the subdermal lymph transport is markedly more efficient than subfascial flow. 2 3

After the disruption of the superficial lymphatic system, as wound healing progresses, scar tissue may develop and restrict the continuous flow of the lymphatic vessels, leading to lymphatic dysfunction 4 and an increased risk of developing posttraumatic lymphedema. 5 6

Due to the importance of the lymphatic system in patients who have suffered severe soft tissue trauma, we described the use of the superficial circumflex iliac artery perforator lymphatic vessels (SCIP-LV) free flap for posttraumatic lymphedema prevention and treatment after important soft tissue damage of critical lymphatic drainage areas. 5 6 Increasing evidence that supports the use of this type of flap has been published, without the need to include lymph nodes (LNs). 7 8 9 10 11 12 13 14 On the other hand, the risk of donor site lymphedema 15 is nil, when performing SCIP-LV.

Although this may be an excellent option in patients requiring a soft tissue reconstruction, there is a group of patients who do not have coverage defects but present with posttraumatic lymphedema. In these cases, the alteration of the lymphatic drainage is due to a scarring process secondary to trauma 16 or to a reconstruction in which restoration of the lymphatic flow was not considered as part of the reconstructive algorithm. 5 6 7 8

The approach to patients with lymphedema secondary to trauma should be personalized, considering the timing of the intervention and the need of soft tissue reconstruction. As a continuation of our previous research, the aim of this study is to propose a comprehensive approach for prevention and treatment of posttraumatic lymphedema using lymphovenous anastomosis (LVA) and lymphatic vessels free flap transfer, reporting our experience in the management of early-stage lymphedema.

## Methods

A retrospective observational study was performed in patients with posttraumatic lymphedema or at risk of developing it, between October 2017 and July 2022, following the principles of the Declaration of Helsinki 17 and approved by the Scientific Ethics Committee (IRB approval number CEC/10/2021). Consent authorizing their procedures was signed by all patients.

**Fig. 1:**
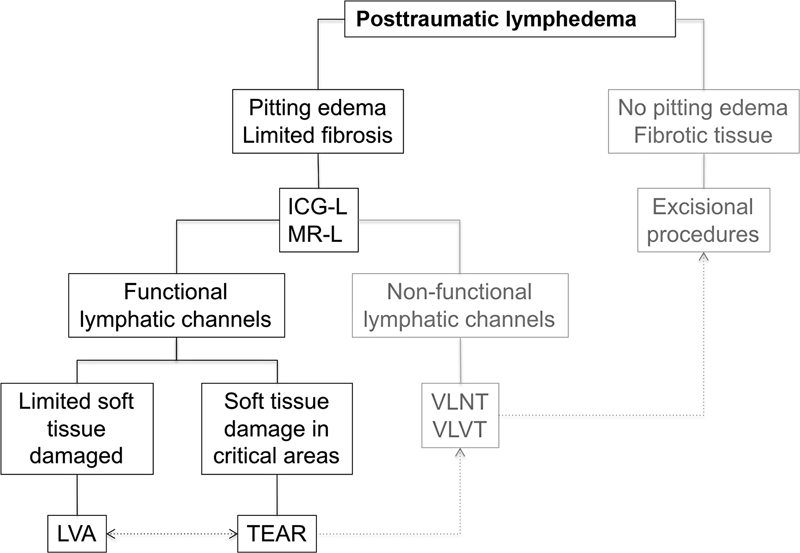
Posttraumatic lymphedema algorithm for surgical treatment. In this study, we include early-stage lymphedema with functional lymphatic channels. ICG-L, indocyanine green lymphography; LVA, lymphovenous anastomosis; MR-L, magnetic resonance lymphangiography; TEAR, total extremity anatomy reconstruction; VLNT, vascularized lymph node transfer; VLVT, vascularized lymphatic vessels transfer; TEAR, total extremity anatomy reconstruction..

Inclusion criteria were patients with early-stage posttraumatic lymphedema (International Society of Lymphology [ISL] stage 1 or early stage 2), lymphatic impairment in indocyanine green lymphography (ICG-L) with functional lymphatic channels in the early phase of this study and edema that does not subside after at least 3 months of complete decongestive therapy (CDT). Patients with nonfunctional lymphatic channels and/or nonpitting edema with fibrotic soft tissue were excluded from this study (ISL late stage 2 or stage 3). Patients with lymphedema, pitting edema, and limited fibrosis underwent to a functional assessment of the lymphatic system with magnetic resonance lymphangiography (MR-L) and ICG-L. ICG-L was performed as follows: 0.1 mL of indocyanine green (5 mg/mL, Diagnostic Green, Aschheim, Germany) was injected subcutaneously into both upper and lower extremities at the first to fourth web spaces of the hands/feet. Immediately (early phase) and 2 hours after the injection (late phase), period in which 30 minutes of supervised exercises were performed, circumferential fluorescent images of lymphatic drainage channels were obtained using a near-infrared camera. Fluorescent images were recorded in real time in photographs and movies. Patients with functional lymphatic channels in early phase were included in this study. Cases with functional lymphatic channels and limited soft tissue damage were proposed for LVA. Patients with functional lymphatic channels and acute or prior soft tissue damage needing skin coverage or replacement were proposed for total extremity anatomy reconstruction (TEAR) as we previously published. 5 6 Patients with nonfunctional lymphatic channels in MR-L and in both the early and late ICG-L phases (stardust or diffuse pattern), but with pitting edema, were candidates for vascularized lymph node transfer (VLNT), or vascularized lymphatic vessels transfer (VLVT), as described by Chen et al. 9 In patients with lymphedema, presenting nonpitting edema and fibrotic soft tissue, excisional procedures were proposed ( Fig. 1 ).

### Surgical Technique

Patients with functional lymphatic channels and limited soft tissue damage were proposed for supermicrosurgical LVA. Preoperatory planning was performed with ICG-L as previously described and using Augmented Reality Microsurgical Planning with a Smartphone for LVA. 18 Findings were marked and photographs were taken in standardized projections. These images were imported to an augmented reality app (Camera Lucida AR, Seattle, WA) to overlap the image with the camera. Markings were performed, guided through the smartphone screen, fixing the image to the extremity landmarks (elbow, wrist, fingers/knee, ankle, toes), identifying the ICG linear pattern lymphatic vessels (early phase) and the dermal backflow (late phase). End-to-end LVA were performed using 11–0 nylon, in the overlapping area of the linear pattern with the dermal backflow, distal to trauma area.

The anteromedial leg, medial aspect of elbow and knee, and medial aspect of arm and thigh are areas with higher lymphatic vessels density. 19 20 In patients with high energy and/or full thickness soft tissue damage greater than 5 cm with lymphatic injury in those critical areas, TEAR was performed to prevent posttraumatic lymphedema. In lymphedema cases with dermal backflow pattern in ICG-L (late phase) and acute injury or scarred tissue, TEAR was performed to treat posttraumatic lymphedema.

**Fig. 2:**
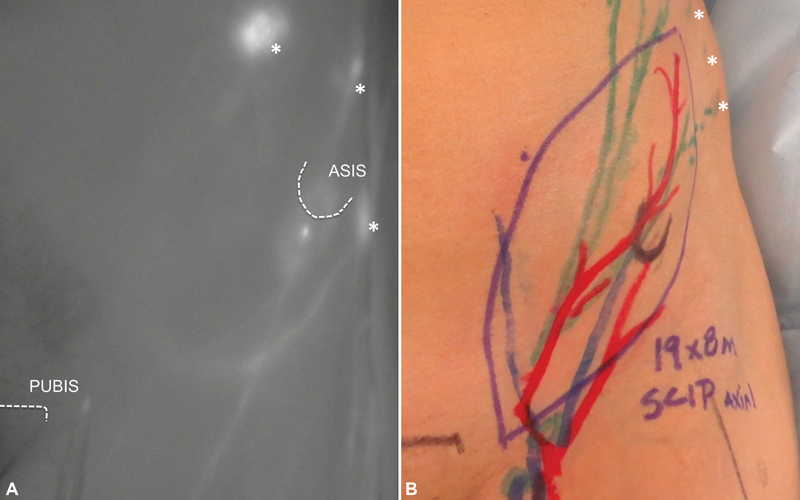
Indocyanine green lymphography (ICG-L) of the inguinal area to study the lymphatic vessels anatomy of the SCIP-LV for preoperative planning. ( **A** ) ICG-L of the groin. ( **B** ) SCIP-LV free flap markings for TEAR approach. The asterisks (*) show ICG injecting sites. ASIS, anterior superior iliac spine; SCIP-LV, superficial circumflex iliac artery perforator lymphatic vessels; TEAR, total extremity anatomy reconstruction.

**Fig. 3:**
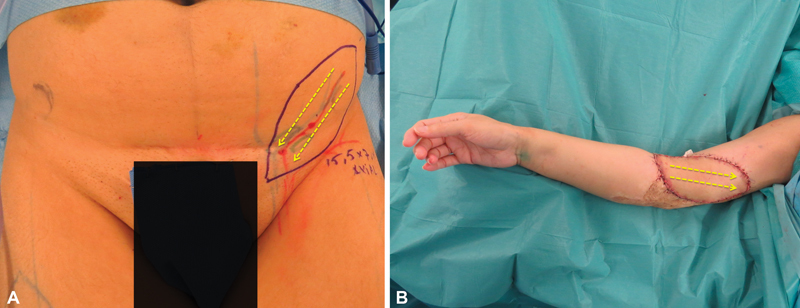
SCIP-LV free flap inset. ( **A** ) The lymphatic system of the groin drains from lateral to medial. ( **B** ) The medial aspect of the flap was placed in a proximal location of the extremity defect and the lateral aspect in a distal location. SCIP-LV, superficial circumflex iliac artery perforator lymphatic vessels.

Same surgeon performed superficial circumflex iliac artery perforator lymphatic vessels free flaps and used for TEAR, as described in our previous reports. 5 6 Preoperative planning was performed by using computed tomography angiography 21 and augmented reality. 22 SCIP flap lymphatic vessels anatomy was studied injecting 0.1 mL of ICG craniolateral to the anterior superior iliac spine and used for designing and performing the inset of the flap, for lymph flow restoration ( Fig. 2 ). The flap was based on the superficial branch of the superficial circumflex iliac artery and harvested including the deep fat layer without LN, by performing a meticulous dissection and keeping them in the donor site. The lateral aspect of the flap was placed distally and the medial aspect in the proximal edge of the injury ( Fig. 3 ).

One end-to-side anastomosis to a main artery or end-to-end perforator-to-perforator anastomosis were performed with 9–0 or 10–0 nylon and one/two veins within the defect were anastomosed. Deep and superficial fat layers of the flap and the recipient site were contacted with 4–0 Vicryl (Ethicon, NJ), and skin was closed with 4–0 nylon.

Soft tissue injuries in lymphatic critical areas were reconstructed using the TEAR approach to prevent posttraumatic lymphedema. ICG-L was performed for lymphatic vessels mapping and marked until the distal edge of the wound at the level of lymphatic disruption. TEAR was performed contacting the flap tissue with healthy recipient soft tissue to provide a lymphatic vessel bridge through the flap. 6

### Postoperative Care

In LVA patients, a postoperative vasodilator mixed with saline was infused continuously for 24 hours, 20 μg/d prostaglandin E1 (Alprostapint; Pint Pharma, Vienna, Switzerland) and 100 mg of acetylsalicylic acid (Cardioaspirine; Bayer, Leverkusen, Germany) was given per day. Prophylactic low-molecular-weight heparin (Clexane; Sanofi-Aventis, Paris, France), 40 mg/d, was injected subcutaneously until ambulation. Compression stockings (30–40 mm Hg) were applied immediately after surgery. Patients were discharged on postoperative day 1 unless there are other medical problems. Flat knit compression stockings were worn immediately after surgery and CDT started 2 weeks after surgery (manual lymph drainage, compression therapy and low-load, nonimpact exercises), once or twice a week depending on patient needs.

In SCIP-LV patients, a postoperative vasodilator mixed with saline was infused continuously for 5 to 7 days, 10 μg/d prostaglandin E1 (Alprostapint; Pint Pharma, Vienna, Switzerland) and 100 mg of acetylsalicylic acid (Cardioaspirine; Bayer, Leverkusen, Germany) was given per day. Prophylactic low-molecular-weight heparin (Clexane; Sanofi-Aventis, Paris, France), 40 mg/d was injected subcutaneously until ambulation. Graduated pressure garments (30–60 mm Hg) were applied from day 5 to 7 to reduce edema and to mold the flap. This also enables early ambulation by day 7 to 10. Patients were discharged from the hospital within 10 to 14 days unless there are other medical problems.

### Clinical Assessment

Quality of life was assessed through a questionnaire 23 24 and measurements of both upper/lower limbs were performed in patients with posttraumatic lymphedema as we previously described, 6 both before and after surgery. Preoperative and postoperative circumference perimeter were taken at different levels with a tape measure—lower extremity: superior edge of the patella, 10 cm above the superior edge of the patella, 10 cm below the inferior edge of the patella, ankle, and mid foot—upper extremity: elbow, 5 cm above and below the elbow, wrist, and mid hand. Extremities volumes were calculated according to the truncated cone Eq, 25 distal to the scars of the trauma where lymphedema was present. Lymphedema improvement after surgery was assessed by calculating the percentage of excess volume (PEV) of the affected limb (PEV = volume of affected limb − volume of unaffected contralateral limb −/− volume of unaffected contralateral limb) and the reduction of excess volume (REV) between preoperative and postoperative (REV = [preoperative PEV − postoperative PEV] −/− preoperative PEV). The follow-up period was at least 12 months with clinical evaluation at 3, 6, and 12 months postoperatively.

## Results

Twenty-eight patients were operated using a comprehensive approach during the study period, both to treat and prevent lymphedema. Mean follow-up period was 20 months (range: 12–36). Twelve patients with posttraumatic lymphedema and limited soft tissue damage (mean age: 48.2 y old [range: 33–62]; mean body mass index [BMI]: 25.8 kg/m ^2^ [range: 23–29]; 12 lower extremity cases), seven patients with posttraumatic lymphedema and scarred or critical soft tissue damage (mean age: 43.5 y old [range: 26–58]; mean BMI: 24.1 kg/m ^2^ [range: 22–27]; three upper and four lower extremities cases), and nine patients with acute soft tissue and lymphatic injury (mean age: 40.4 y old [range: 22–53]; mean BMI: 24.6 kg/m ^2^ [range: 21–27]; two upper and seven lower extremity cases).

Posttraumatic lymphedema was present in 27 patients. Nineteen patients met the inclusion criteria.

### Lymphovenous Anastomosis

**Fig. 4:**
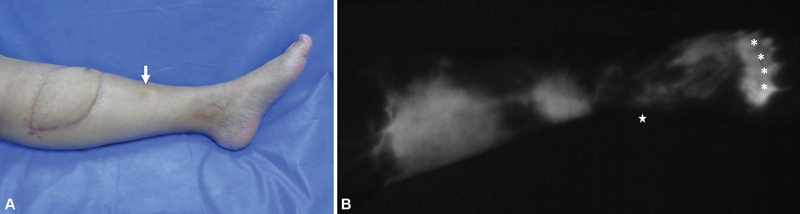
Nonlymphatic reconstruction with an ALT flap without considering the axiality of the lymphatic drainage. ( **A** ) Left leg lymphedema distal to ALT flap. ( **B** ) Lymphatic dysfunction assessed with ICG-L. The arrow indicates pitting edema, the asterisks (*) show ICG injecting site in foot web spaces, and the star indicates the medial malleolus. ALT, anterolateral thigh flap; ICG-L, indocyanine green lymphography.

**Fig. 5:**
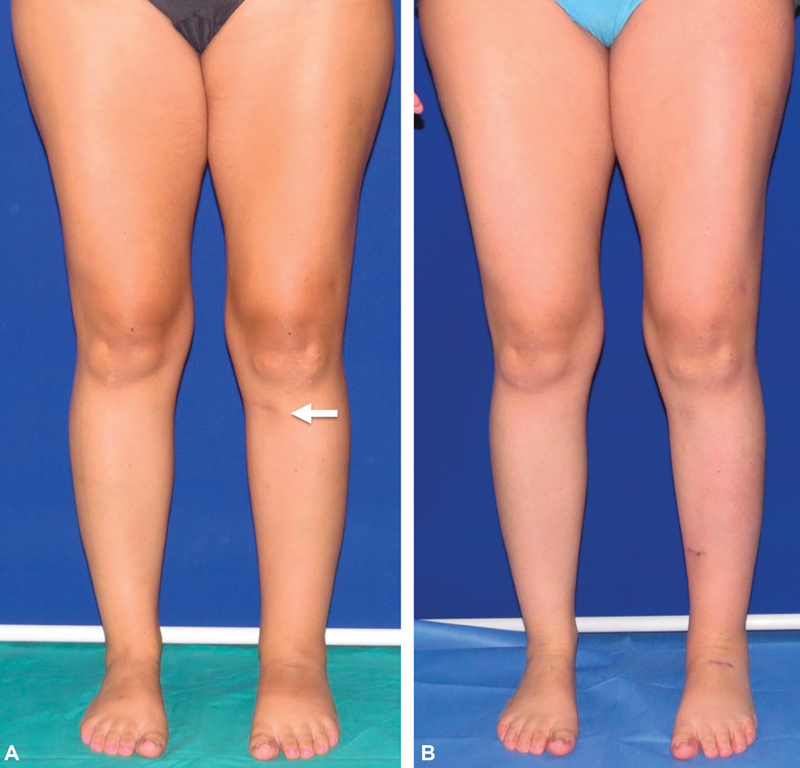
A 28-year-old female patient with 2 years of left lower limb posttraumatic lymphedema after an orthopaedic surgery treated with three lymphovenous anastomosis. ( **A** ) Preoperative excess volume (PEV) was 8.68%. ( **B** ) Twelve months after surgery, PEV was 0.12% and the reduction of excess volume was 98.54% (Patient 1 in Table 1 ).

**Fig. 6:**
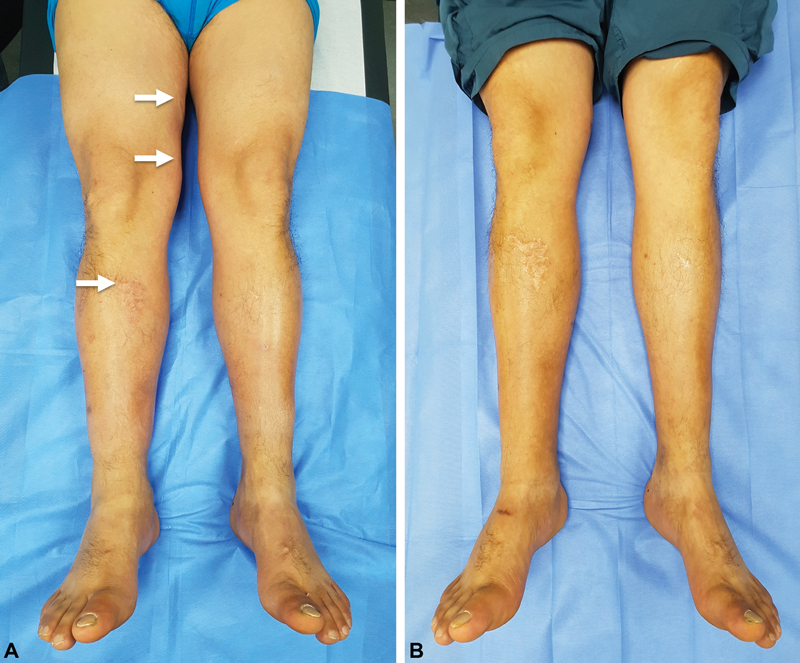
A 58-year-old male patient with 6 years of right lower limb posttraumatic lymphedema after a tiger attack treated with three lymphovenous anastomosis. ( **A** ) Preoperative excess volume (PEV) was 13.35%. ( **B** ) Thirteen months after surgery, PEV was 7.37% and the reduction of excess volume was 44.7% (Patient 3 in Table 1 ).

Twelve patients had limited soft tissue damage secondary to fasciotomies (4), orthopaedic surgery (2), nonlymphatic reconstruction (5), and tiger attack (1). Nonlymphatic reconstruction refers to soft tissue reconstruction with a flap without considering the axiality of the lymphatic drainage ( Fig. 4 ) or using skin graft. ICG-L was performed as described previously, and an average of three LVA were performed in the overlapping area between linear and dermal backflow pattern, in each patient (mean operation time: 120 min [range: 70–200]). End-to-end LVA configuration was preferred in all cases and always distal to the scars. Mean preoperative PEV was 9.02%. Postoperative PEV was 3.73% and mean REV was 58.82% at 12-month follow-up ( Table 1 ). Quality of life improved importantly after surgery, from a preoperative score of 67/100 to a postoperative score of 34/100 (improvement of 49.25%; Figs 5 and 6 ).

**Table 1:** Lymphovenous anastomosis cases

	Gender	Extremity	Trauma	No. of LVA	PEV pre-op (%)	LEQoLiS pre-op	PEV post-op (%)	LEQoLiS post-op	REV (%)
1	Female	Lower	Orthopaedic surgery	3	8.68	86/100	0.12	57/100	98.54
2	Female	Lower	Orthopaedic surgery	4	7.03	71/100	3.58	6/100	49.07
3	Male	Lower	Tiger attack	3	13.35	58/100	7.37	36/100	44.7
4	Male	Lower	Fasciotomies	4	12.78	50/100	3.57	31/100	72.04
5	Female	Lower	Free flap	3	4.4	78/100	2.55	65/100	41.98
6	Female	Lower	Fasciotomies	2	7.25	66/100	(−) 1.78	5/100	124.63
7	Female	Lower	Fasciotomies	2	8.31	59/100	7.13	38/100	14.23
8	Male	Lower	Propeller flap	3	8.6	66/100	4.82	32/100	43.95
9	Male	Lower	Grafted degloving injury	3	14.2	69/100	7.12	33/100	49.85
10	Female	Lower	Free flap	2	8.5	65/100	3.56	35/100	58.11
11	Female	Lower	Free flap	2	8.12	70/100	2.6	34/100	67.98
12	Female	Lower	Fasciotomies	2	7.1	66/100	4.2	33/100	40.84
			Mean	2.75	9.02	67/100	3.73	34/100	58.82

Abbreviations: LEQoLiS, Lymphedema Quality of Life Score; LVA, lymphovenous anastomosis; PEV, percentage of excess volume; post-op, postoperative; pre-op, preoperative; REV, reduction of excess volume; SCIP-LV, superficial circumflex iliac artery perforator lymphatic vessels.

### Therapeutic Total Extremity Anatomy Reconstruction

**Fig. 7:**
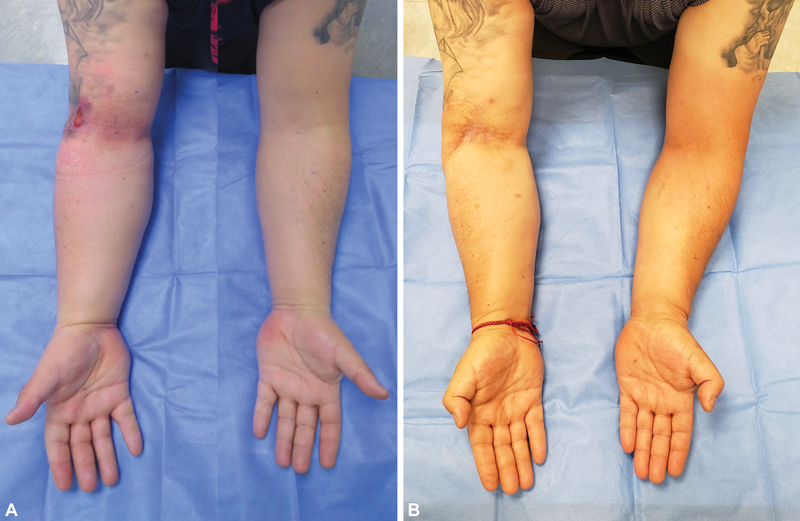
Case 3. ( **A** ) Preoperative picture. ( **B** ) Twenty-month postoperative picture. A 42-year-old male patient with 10 months of right upper limb posttraumatic lymphedema and chronic ulcer after a degloving injury treated with local flaps. Scarred tissue was excised and reconstruction was performed with TEAR approach using SCIP-LV. ( **A** ) Preoperative excess volume (PEV) was 19.25%; ( **B** ) Twenty months after surgery, PEV was 5.55% and the reduction of excess volume was 71.17% (patient 2 in Table 2 ). SCIP-LV, superficial circumflex iliac artery perforator lymphatic vessels; TEAR, total extremity anatomy reconstruction.

Seven patients had scarred or critical soft tissue damage secondary to degloving injuries (4), open tibial fracture (2), and necrotizing fasciitis (1). ICG-L was performed and lymphatic vessels were mapped until the edge of the healthy skin. Scarred tissue excision, flap tailoring to the defect, vascular anastomosis, and inset of the flap contacting healthy tissue considering the lymphatic flow direction were performed (mean operation time: 315 min [range: 280–420]). There were no flap failures. Mean preoperative PEV was 19.49%. Postoperative PEV was 7.76% and mean REV was 58.77% at 12-month follow-up ( Table 2 ). Quality of life improved importantly after surgery, from a preoperative score of 55/100 to a postoperative score of 27/100 (improvement of 50.9%; Fig. 7 ).

**Table 2:** Therapeutic total extremity anatomy reconstruction cases

	Gender	Extremity	Trauma	Reconstruction	PEV pre-op (%)	LEQoLiS pre-op	PEV post-op (%)	LEQoLiS post-op	REV (%)
1	Female	Upper	Degloving injury	SCIP-LV free flap	27.86	66/100	12.38	33/100	55.6
2	Male	Upper	Degloving injury	SCIP-LV free flap	19.25	50/100	5.55	22/100	71.17
3	Female	Upper	Necrotizing Fasciitis	SCIP-LV free flap	10.46	42/100	6.44	18/100	38.42
4	Male	Lower	Degloving injury	SCIP-LV free flap	20.12	60/100	(-) 1.35	25/100	106.46
5	Female	Lower	Open tibial fracture	SCIP-LV free flap	18.74	52/100	10.6	30/100	43.44
6	Male	Lower	Degloving injury	SCIP-LV free flap	19.55	58/100	11.5	32/100	41.18
7	Male	Lower	Open tibial fracture	SCIP-LV free flap	20.5	56/100	9.2	26/100	55.12
				Mean	19.49	55/100	7.76	27/100	58.77

Abbreviations: LEQoLiS, Lymphedema Quality of Life Score; PEV, percentage of excess volume; post-op, postoperative; pre-op, preoperative; REV, reduction of excess volume; SCIP-LV, superficial circumflex iliac artery perforator lymphatic vessels.

### Preventive Total Extremity Anatomy Reconstruction

**Fig. 8:**
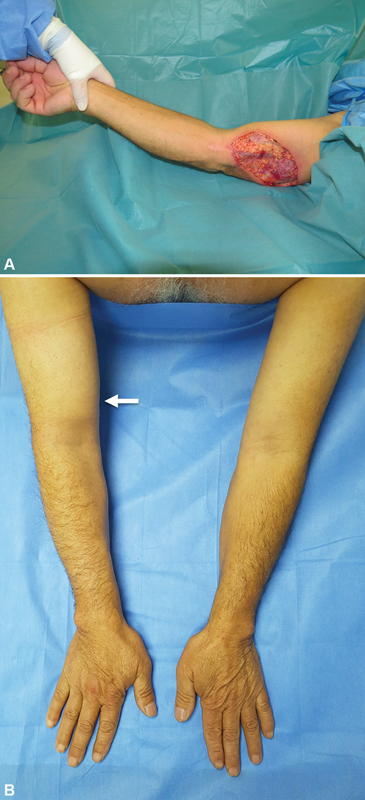
A 52-year-old male patient with right arm injury in a critical lymphatic area secondary to spider bite. ( **A** ) Necrotic tissue was excised and a preventive TEAR approach using SCIP-LV was performed. ( **B** ) Fourteen months after surgery, no lymphedema is seen on the right upper limb (patient 2 in Table 3 ). The arrow indicates SCIP flap location. SCIP-LV, superficial circumflex iliac artery perforator lymphatic vessels; TEAR, total extremity anatomy reconstruction

Nine patients with acute injury in lymphatic critical areas (degloving; open tibial fracture; skin necrosis secondary to spider bite [cutaneous loxoscelism]) underwent to ICG-L study and lymphatic vessels were mapped until the distal edge of the wound at the level of lymphatic disruption. Excision of devitalized soft tissue, flap tailoring to the defect, perforator-to-perforator anastomosis, and inset of the flap contacting healthy tissue considering the lymphatic flow direction were performed (mean operation time: 290 min [range: 250–400]). No lymphedema was detected after at least 1-year follow-up, defined clinically as an increase of the volume with pitting edema of the limb, associated with alterations in the lymphography ( Table 3 ; Fig. 8 ).

**Table 3:** Preventive total extremity anatomy reconstruction cases

	Gender	Extremity	Trauma	Reconstruction	Lymphedema	Follow-up (mo)
1	Male	Upper	Degloving injury	SCIP-LV free flap	No	25
2	Male	Upper	Spider bite	SCIP-LV free flap	No	18
3	Male	Lower	Degloving injury	SCIP-LV free flap	No	36
4	Female	Lower	Open tibial fracture	SCIP-LV free flap	No	21
5	Male	Lower	Open tibial fracture	SCIP-LV free flap	No	19
6	Male	Lower	Open tibial fracture	SCIP-LV free flap	No	18
7	Male	Lower	Open tibial fracture	SCIP-LV free flap	No	15
8	Male	Lower	Open tibial fracture	SCIP-LV free flap	No	12
9	Male	Lower	Degloving injury	SCIP-LV free flap	No	12

Abbreviation: SCIP-LV, superficial circumflex iliac artery perforator lymphatic vessels.

## Discussion

This study revealed the importance of considering the lymphatic system as a relevant part of the decision-making algorithm for limb reconstruction to provide an optimal treatment. Currently, we can prevent the occurrence of posttraumatic lymphedema by using an approach to restore lymphatic drainage at the time of reconstruction. On the other hand, if lymphedema is clinically manifest, we must assess whether there is soft tissue damage to establish the need to reconstruct that aspect or the problem is only distal to the scars. With all this in mind, we propose a comprehensive approach: a Post-Traumatic Lymphedema Algorithm for Surgical Treatment ( Fig. 1 ) to prevent and treat post-traumatic lymphedema.

The disruption of the superficial lymphatic drainage system increases the risk of developing posttraumatic lymphedema. In upper and lower extremity, major lymphatic pathways are located along the anteromedial surface of the arm and leg, going toward the axillary and inguinal LNs, respectively. 19 20

Posttraumatic extremity lymphedema incidence is unknown and the problem is underdiagnosed. 26 It is demonstrated that after limb trauma, even without physical disruption of the superficial lymphatic system, 100% of the patients can experience edema due to increased lymphatic production from local inflammation, which is thought to be a contributing mechanism. There is a group of patients in which the edema resolves spontaneously, probably due to a remitted inflammatory process rather than resolved lymphatic dysfunction. It was believed that venous thrombotic disease was a major contributor to post-traumatic edema; however, only 24% of patients presented this problem. 16 On the other hand, lymphatic system interruption with dermal backflow was observed in the affected areas of patients with extensive soft tissue damage and multiple surgeries. 27 28 In the same line, Lohrmann et al 29 evaluated posttraumatic edema with MR-L demonstrating that 25% of limbs presented signs of lymphatic outflow obstruction. The main difference in the management of these patients compared with post oncologic patients is the importance of suspicion for early diagnosis and timely treatment. In addition, there is usually significant soft tissue damage that needs to be considered for reconstruction.

In a study about the lymphatic response after reconstruction to high-energy trauma to the leg, van Zanten et al 4 reported nonfunctional lymphatic vessels in the free flaps of the study sample at an average of 36 months after their soft tissue reconstruction. In local reconstruction and free flap reconstruction groups it was evident that scar tissue blocked the continuous flow of superficial lymphatics and seemed to cause local dermal backflow patterns. This demonstrates that the use of muscle flaps and not considering the lymphatic system at the time of soft tissue reconstruction can increase and risk of developing lymphedema in the long term. Unfortunately, there are no comparative studies to draw conclusions, but we have seen this problem in some cases where local, regional, or free flaps are used, where lymphatic drainage was not considered ( Fig. 4 ). There is increasing evidence to support the use lymphatic vessels free flap without including LN 5 6 7 8 9 10 11 12 13 14 ; on the other hand, there are reports of donor site lymphedema after groin lymph node transfer, 15 which is avoided when performing SCIP-LV. Generally, these studies report the use of this flap to prevent or treat lymphedema secondary to cancer treatment, using it in a free 7 8 9 10 11 or pedicled 12 13 14 fashion. But, in our opinion when performing a soft tissue reconstruction after trauma in a critical area of the lymphatic drainage, the use of flaps rich in lymphatic vessels should be considered and the inset performed following the axiality of the drainage, the same as we and other authors have proposed. 5 8 In this way, it is possible to restore the lymphatic drainage of the limb without LN transfer or supermicrosurgical lymphatic anastomosis.

We consider that the SCIP-LV flap is one of the ideal choices for soft tissue reconstruction of a traumatized limb in which we are planning to either treat or prevent posttraumatic lymphedema because the abundance of lymphatic vessels and the lymph flow direction parallel to its longer axis. Therefore, it is very important to know the lymphatic features of different flaps that eventually could be used to cover a defect, to choose one that is not only rich in lymphatic vessels, but also has the proper axiality of its drainage. This would allow a correct inset to recover the local lymphatic system flow.

Lymphedema usually develops secondary to trauma, distal to the scars, without there necessarily being or having been a soft tissue defect. In these situations, we consider that the ideal procedure is LVA distal to the zone of injury, to perform a bypass that allows decongesting the affected limb. We used ICG-L to diagnose lymphatic dysfunction and to plan the location to perform LVA. 30 31 By performing the ICG-L study in two stages, we can assess the area of functional lymphatic vessels (linear pattern) in the early phase and the areas of dysfunction (dermal backflow) in the late phase. We consider that the overlapping zone between the linear pattern and the dermal backflow is the ideal area to perform LVA since is the area where the dysfunction can be treated.

The greater PEV in patients who underwent to TEAR compared with those who underwent LVA is mainly due to the fact that in the latter patients presented more distal scars, and therefore distal lymphedema (generally at the level of the ankle), so the excess of volume was less compared with a healthy limb. However, the REV in both cases was similar, as was the improvement in quality of life.

Although our series is an initial experience on the treatment of posttraumatic lymphedema at an early stage, it is the first report proposing an individualized approach to trauma-related lymphedema considering the functionality of the lymphatic system and the type of injury. Undoubtedly, more studies and longer follow-up are necessary to establish a definitive treatment, but given the existing evidence and our experience, we propose this comprehensive approach to prevent or treat posttraumatic lymphedema. We believe this algorithm should be the new standard of care in limb reconstruction, providing restoration of the damaged lymphatic system to prevent or treat posttraumatic lymphedema, rather than just resolving the coverage defect.
